# Tele-ophthalmology

**DOI:** 10.4103/0974-620X.48425

**Published:** 2009

**Authors:** Pukhraj Rishi

**Affiliations:** Shri Bhagwan Mahavir Vitreoretinal Services, Sankara Nethralaya, Chennai, India

A new aspect in medical care
‘Telemedicine’ is up here.
Taking the doctor to the patients‘ doorstep
Is indeed a novel concept.
A satellite van for a screening camp
Is fitted with ‘things’, including slit-lamp.
Live images are captured and transmitted
Through a satellite band that is committed
Exclusively for a two-way conference
Which reduces a long distance.
(If you don‘t have access to satellite
ISDN connection is alright)
Images are received in a spurt
And then read by an expert.
Opinion from the base hospital
Would aver on the need for referral.
Patients with diverse conditions
Find themselves in a position
To be seeing a specialist
Or being dispensed by an Optometrist.
It also serves as a screening methodology
In the study of disease epidemiology.
The sequence thus works in remote sectors
Offsetting the climatic and geographical factors.
Prisons and Orphanages are potential beneficiaries
Maybe someday convents and monasteries.
Doctors can also seek an opinion
For entities they see, one in a million.
Telemedicine is effective and it saves
‘Tis medical care on the ‘waves’!

